# The NF-κB signalling pathway in colorectal cancer: associations between dysregulated gene and miRNA expression

**DOI:** 10.1007/s00432-017-2548-6

**Published:** 2017-11-29

**Authors:** Martha L. Slattery, Lila E. Mullany, Lori Sakoda, Wade S. Samowitz, Roger K. Wolff, John R. Stevens, Jennifer S. Herrick

**Affiliations:** 1 Department of Medicine, University of Utah, 383 Colorow, Salt Lake City, UT 84108 USA; 2 Division of Research, Kaiser Permanente Northern California, Oakland, CA USA; 3 Department of Pathology, University of Utah, Salt Lake City, UT USA; 4 Department of Mathematics and Statistics, Utah State University, Logan, UT USA

**Keywords:** NF-κB, Colorectal cancer, MiRNA, mRNA, *TNFSFR11A*

## Abstract

**Background:**

The nuclear factor-kappa B (NF-κB) signalling pathway is a regulator of immune response and inflammation that has been implicated in the carcinogenic process. We examined differentially expressed genes in this pathway and miRNAs to determine associations with colorectal cancer (CRC).

**Methods:**

We used data from 217 CRC cases to evaluate differences in NF-κB signalling pathway gene expression between paired carcinoma and normal mucosa and identify miRNAs that are associated with these genes. Gene expression data from RNA-Seq and miRNA expression data from Agilent Human miRNA Microarray V19.0 were analysed. We evaluated genes most strongly associated and differentially expressed (fold change (FC) of > 1.5 or < 0.67) that were statistically significant after adjustment for multiple comparisons.

**Results:**

Of the 92 genes evaluated, 22 were significantly downregulated and nine genes were significantly upregulated in all tumours. Two additional genes (*CD14* and *CSNK2A1*) were dysregulated in MSS tumours and two genes (*CARD11* and *VCAM1*) were downregulated and six genes were upregulated (*LYN, TICAM2, ICAM1, IL1B, CCL4* and *PTGS2*) in MSI tumours. Sixteen of the 21 dysregulated genes were associated with 40 miRNAs. There were 76 miRNA:mRNA associations of which 38 had seed-region matches. Genes were associated with multiple miRNAs, with *TNFSRF11A* (*RANK*) being associated with 15 miRNAs. Likewise several miRNAs were associated with multiple genes (miR-150-5p with eight genes, miR-195-5p with four genes, miR-203a with five genes, miR-20b-5p with four genes, miR-650 with six genes and miR-92a-3p with five genes).

**Conclusions:**

Focusing on the genes and their associated miRNAs within the entire signalling pathway provides a comprehensive understanding of this complex pathway as it relates to CRC and offers insight into potential therapeutic agents.

**Electronic supplementary material:**

The online version of this article (10.1007/s00432-017-2548-6) contains supplementary material, which is available to authorised users.

## Introduction

The nuclear factor-kappa B (NF-κB) signalling pathway is a key regulator of inflammation and has been associated with carcinogenesis (Merga et al. 2016). The NF-κB family comprised five hetero or homodimers: RelA (p65), RelB, NF-κB1 (p50 and its precursor p105), NF-κB2 (p52 and its precursor p100), and c-Rel (Pereira and Oakley 2008). In an inactive state, NF-κB dimers are bound to inhibitors of NF-κB (IκB) [such as inhibitory κB kinases (IKK) or their inactive precursors (i.e. p105 and p100)]. The classical or canonical NF-κB signalling pathway is activated by cytokines, such as IL-1 and TNF, T-cell receptors (TCR), or B-cell receptors (BCR) which stimulate the IKK complex, phosphorylating p105 which releases the NF-κB dimers to the nucleus where they activate gene expression. Three IKKs, IKKα, IKKβ and IKKγ or NEMO (NF-κB essential modulator), are involved in the canonical pathway. The alternative pathway (non-canonical pathway) originates through B-cell activation factor (BAFF-R), lymphotoxin β-receptor (LTβR), CF40 receptor activator for nuclear factor-kappa B (RANK), and TNFR2, which in turn activate adaptor protein NF-κB-inducing kinase (NIK) which activates IKKα. IKKα activity induces phosphorylation of RelB and p100. It has been proposed that the canonical pathway is associated with an acute phase response and is integrated with the non-canonical pathway for a more sustained immunological response (Merga et al. 2016). In colon cancer specifically, IKKB-induced NF-κB activation in intestinal epithelial cells and its corresponding inflammation appears to have an essential role in tumour formation (Slattery and Fitzpatrick 2009).

NF-κB is a transcription factor which, when activated can regulate over 200 genes involved in inflammation and cell function and survival (Pereira and Oakley 2008). MiRNAs, small non-coding RNAs that bind to the 3′UTR of the protein-coding mRNAs and inhibit their translation, may also be transcriptional targets. This dynamic provides a mechanism for dysregulation of genes through the activation of transcription factors such as NF-κB (Hoesel and Schmid 2013). Several miRNAs, including let-7, miR-9, miR-21, miR-143, miR-146 and miR-224, are transcriptional targets of NF-κB (Hoesel and Schmid 2013). These miRNAs have been shown to be involved in feedback mechanisms that influence the NF-κB signalling pathway by either targeting upstream signalling molecules or members of the NF-κB family. Let-7 has been shown to target IL-6 in that a reduction in Let-7 results in higher levels of IL-6 and activation of NF-κB in a positive feedback loop (Iliopoulos et al. 2009). It has been suggested that miR-520e may modify cell proliferation by targeting NIK (Zhang et al. 2012). Other studies have shown that miR-15a, miR-16 and miR-223 can influence IKKα protein expression (Li et al. 2010). While these data support the co-regulatory functions of NF-κB and miRNAs, there has not been a systematic evaluation of the NF-κB signalling pathway as it relates to miRNAs.

There is strong evidence to suggest that inflammation is a key element in the etiology of colorectal cancer (CRC) [reviewed in (Slattery and Fitzpatrick 2009)]. In this paper we examine expression levels of 92 genes in the NF-κB signalling pathway. We focus on genes within this signalling pathway that are dysregulated, in that the expression levels in the tumours are statistically different than in normal mucosa with a fold change (FC) of difference that is > 1.50 or < 0.67. We examine differential expression of miRNAs with dysregulated pathway genes. Our aim is to broaden our understanding of the NF-κB signalling pathway as it relates to CRC.

## Methods

### Study participants

Study participants come from two population-based case–control studies that included all incident colon and rectal cancer patients diagnosed between 30 and 79 years of age in Utah or who were members of Kaiser Permanente Northern California (KPNC). Participants were non-Hispanic white, Hispanic or black for the colon cancer study; the rectal cancer study also included people of Asian race (Slattery et al. 1997, 2003). Cases were verified by tumour registry as a first primary adenocarcinoma of the colon or rectum, diagnosed between October 1991 and September 1994 (colon study) and between May 1997 and May 2001 (rectal study) (Slattery et al. 2016). The Institutional Review Boards at the University of Utah and at KPNC approved the study.

### RNA processing

Methods for RNA processing and mRNA and miRNA analysis have been previously described and are summarised here (Slattery et al. 2017a, b). Formalin-fixed paraffin-embedded tissue from the initial biopsy or surgery was used to extract RNA. RNA was extracted, isolated and purified from carcinoma tissue and adjacent normal mucosa as previously described (Slattery et al. 2015). We observed no differences in RNA quality based on age of the tissue.

### mRNA:RNA-seq sequencing library preparation and data processing

Total RNA from 245 colorectal carcinoma and normal mucosa pairs was chosen for sequencing based on availability of RNA and high quality miRNA data in order to have both mRNA and miRNA from the same individuals; the 217 pairs that passed quality control (QC) were used in these analyses (Pellatt et al. 2016a, b). RNA library construction was performed with the Illumina TruSeq Stranded Total RNA Sample Preparation Kit with Ribo-Zero. The samples were then fragmented and primed for cDNA synthesis, adapters were then ligated onto the cDNA and the resulting samples were then amplified using PCR; the amplified library was then purified using Agencourt AMPure XP beads. A more detailed description of the methods can be found in our previous work (Slattery et al. 2015a, b). Illumina TruSeq v3 single-read flow cell and a 50 cycle single-read sequence run were performed on an Illumina HiSeq instrument. Reads were aligned to a sequence database containing the human genome (build GRCh37/hg19, February 2009 from genome.ucsc.edu) and alignment was performed using novoalign v2.08.01. Total gene counts were calculated for each exon and UTR of the genes using gene coordinates obtained from http://genome.ucsc.edu. Genes that were not expressed in our RNA-Seq data or for which the expression was missing for the majority of samples were excluded from further analysis (Slattery et al. 2015a, b).

### miRNA

The Agilent Human miRNA Microarray V19.0 was used. Data were required to pass QC parameters established by Agilent that included tests for excessive background fluorescence, excessive variation among probe sequence replicates on the array, and measures of the total gene signal on the array to assess low signal. Samples failing to meet quality standards were relabelled, hybridised to arrays and re-scanned. If a sample failed QC assessment a second time, the sample was excluded from analysis. The repeatability associated with this microarray was extremely high (*r* = 0.98) (Slattery et al. 2016); comparison of miRNA expression levels obtained from the Agilent microarray to those obtained from qPCR had an agreement of 100% in terms of directionality of findings and the FCs were almost identical (Pellatt et al. 2016). To normalise differences in miRNA expression that could be attributed to the array, amount of RNA, location on array, or factors that could erroneously influence miRNA expression levels, total gene signal was normalised by multiplying each sample by a scaling factor which was the median of the 75th percentiles of all the samples divided by the individual 75th percentile of each sample (Agilent GeneSpring User Manual).

### *NF-κB *signalling genes

The Kyoto Encyclopedia of Genes and Genomes (KEGG) (http://www.genome.jp/kegg-gin/show_pathway?hsa04064) Pathway map for NF-Kappa B Signalling was used to identify genes. We identified 95 genes (Supplemental Table 1) in this signalling pathway; 92 of these genes had sufficient expression in our CRC tissue for further analysis.

### Statistical methods

We utilised negative binomial mixed effects model in SAS (accounting for carcinoma/normal status as well as subject effect) to determine which genes in the NF-κB signalling pathway had a significant difference in expression between individually paired CRC and normal mucosa and their related fold changes (FC). In the negative binomial model we offset the overall exposure as the log of the expression of all identified protein-coding genes (*n* = 17,461). The Benjamini and Hochberg (Benjamini 1995) procedure was used to control the false discovery rate (FDR) using a value of < 0.05. A FC greater than one indicates a positive differential expression (i.e. upregulated in carcinoma tissue) while a FC between zero and one indicates a negative differential expression (i.e. downregulated in carcinoma tissue). We calculated the level of expression of each gene by dividing the total expression for that gene in an individual by the total expression of all protein-coding genes per million transcripts (RPMPCG or reads per million protein-coding genes). We considered overall CRC differential expression as well as differential expression for microsatellite unstable (MSI) and stable (MSS) tumours separately.

We arbitrarily focused on those genes and miRNAs with FCs of > 1.50 or < 0.67 in order to have more meaningful biological differences between carcinoma and normal samples. There were 814 miRNAs expressed in greater than 20% of normal colorectal mucosa samples that were analysed; differential expression was calculated using subject-level paired data as the expression in the carcinoma tissue minus the expression in the normal mucosa. In these analyses, we fit a least squares linear regression model to the RPMPCG differential expression levels and miRNA differential expression levels. P-values were generated using the bootstrap method by creating a distribution of 10,000 F statistics derived by resampling the residuals from the null hypothesis model of no association between gene expression and miRNA expression using the boot package in R. Linear models were adjusted for age and sex. Multiplicity adjustments for mRNA:miRNA associations were made at the gene level using the FDR by Benjamini and Hochberg (Benjamini 1995).

### Bioinformatics analysis

We analysed miRNAs and targeted mRNAs for seed-region matches. The mRNA 3′ UTR FASTA as well as the seed-region sequence of the associated miRNA were analysed to determine seed-region pairings between miRNA and mRNA. MiRNA seed regions were calculated as described in our previous work (Mullany et al. 2016); we calculated and included seeds of six, seven and eight nucleotides in length. Our hypothesis is that a seed match would increase the likelihood that identified genes associated with a specific miRNA were more likely to have a direct association given a higher propensity for binding. As miRTarBase (Chou et al. 2016) uses findings from many different investigations spanning across years and alignments, we used FASTA sequences generated from both GRCh37 and GRCh38 Homo sapiens alignments, using UCSC Table browser (https://genome.ucsc.edu/cgi-bin/hgTables) (Karolchik et al. 2004). We downloaded FASTA sequences that matched our Ensembl IDs and had a consensus coding sequences (CCDS) available. Analysis was done using scripts in R 3.2.3 and in perl 5.018002.

## Results

The majority of cases were located in the colon (77.9%) rather than the rectum (22.1%) (Table 1).

**Table 1:** Description of study population

	*N*	%
Site		
Colon	169	77.9
Rectal	48	22.1
Sex		
Male	118	54.4
Female	99	45.6
Age		
Mean (SD)	64.8	10.1
Race		
Non-Hispanic White	161	74.2
Hispanic	14	6.5
Non-Hispanic Black	8	3.7
Unknown	34	15.7
Tumour phenotype		
*TP53* mutated	103	47.5
*KRAS* mutated	69	31.8
*BRAF*-mutated	21	10.1
CIMP High	45	20.7
MSI	29	13.4

The mean age of the study population was 64.8 years and 54.4% of the population were male. Non-Hispanic white cases were the predominate race (74.2%) followed by Hispanic (6.5%) and Black (3.7%). The majority of tumours (86.6%) were considered microsatellite stable while 13.4% had MSI.

Twenty-two genes were significantly downregulated when considering a FC of < 0.67 and nine genes were significantly upregulated with a FC of > 1.50 (Table 2).

**Table 2:** Associations between paired carcinoma and normal genes expression in the KEGG NF-κB signalling pathway

Gene name	Tumour mean	Normal mean	Fold change	Raw *P* value	FDR *P* value
*CCL13*	0.71	6.30	0.11	1.70E−23	8.68E−23
*CCL19*	1.02	7.03	0.14	7.29E−23	3.53E−22
*PRKCB*	14.65	53.12	0.28	1.52E−41	3.49E−40
*TNFRSF13C*	1.72	5.88	0.29	1.43E−16	4.88E−16
*CD40LG*	1.00	3.33	0.30	9.05E−17	3.33E−16
*CXCL12*	23.04	74.27	0.31	7.44E−49	2.28E−47
*PLCG2*	16.91	52.18	0.32	1.31E−36	2.00E−35
*BTK*	5.16	15.34	0.34	4.95E−25	2.87E−24
*CCL21*	5.84	16.77	0.35	4.21E−16	1.38E−15
*BCL2*	24.42	62.11	0.39	3.73E−39	6.86E−38
*ZAP70*	7.77	18.76	0.41	1.51E−21	6.95E−21
*LTB*	3.10	7.26	0.43	3.88E−13	1.11E−12
*BLNK*	20.68	47.50	0.44	2.98E−27	2.11E−26
*TNFRSF11A*	53.68	118.87	0.45	2.30E−21	1.01E−20
*LTA*	0.61	1.26	0.48	1.13E−04	2.00E−04
*LBP*	0.15	0.28	0.53	3.39E−02	4.72E−02
*LAT*	7.72	12.01	0.64	6.19E−10	1.54E−09
*CD40*	16.05	24.91	0.64	1.97E−08	4.31E−08
*IL1R1*	57.71	89.40	0.65	9.55E−17	3.38E−16
*TNFSF14*	6.37	9.76	0.65	3.20E−06	6.01E−06
*MAP3K14*	33.86	51.73	0.65	1.46E−24	7.90E−24
*BIRC3*	79.53	120.36	0.66	1.83E−14	5.42E−14
*CD14*	9.64	14.13	0.68	1.36E−07	2.84E−07
*CFLAR*	203.49	290.53	0.70	2.89E−29	2.42E−28
*RIPK1*	49.35	69.68	0.71	1.19E−19	4.57E−19
*LY96*	0.91	1.25	0.73	9.16E−02	1.15E−01
*TNFRSF1A*	90.65	118.74	0.76	4.32E−20	1.81E−19
*ATM*	239.32	312.70	0.77	4.82E−16	1.53E−15
*BCL10*	42.63	55.30	0.77	4.62E−09	1.06E−08
*NFKBIA*	58.77	75.03	0.78	1.13E−07	2.41E−07
*LCK*	8.55	10.61	0.81	2.13E−02	3.06E−02
*TIRAP*	21.76	26.81	0.81	3.76E−06	6.91E−06
*PIAS4*	29.27	35.75	0.82	6.64E−07	1.33E−06
*BIRC2*	81.92	98.12	0.83	6.71E−10	1.63E−09
*PRKCQ*	7.27	8.69	0.84	3.63E−02	4.99E−02
*VCAM1*	13.23	15.65	0.85	3.98E−02	5.37E−02
*RELB*	24.80	28.79	0.86	1.24E−03	1.96E−03
*TNFSF13B*	4.40	5.11	0.86	9.98E−02	1.24E−01
*TRADD*	25.11	28.92	0.87	2.47E−03	3.79E−03
*TAB2*	128.75	146.44	0.88	8.45E−07	1.65E−06
*TAB1*	29.01	32.68	0.89	1.36E−03	2.13E−03
*TRAF1*	46.52	51.87	0.90	1.55E−02	2.26E−02
*TRIM25*	136.97	150.69	0.91	2.32E−04	3.89E−04
*ERC1*	140.89	154.24	0.91	2.57E−03	3.88E−03
*NFKB2*	45.18	49.11	0.92	5.89E−02	7.74E−02
*NFKB1*	77.63	83.98	0.92	7.65E−03	1.14E−02
*CARD10*	73.25	78.85	0.93	7.35E−02	9.39E−02
*PIDD*	28.99	31.19	0.93	1.08E−01	1.33E−01
*DDX58*	45.26	48.56	0.93	1.77E−01	2.06E−01
*IKBKB*	137.36	145.80	0.94	6.63E−02	8.59E−02
*TNF*	1.95	2.04	0.96	7.52E−01	7.60E−01
*PTGS2*	16.66	17.36	0.96	6.99E−01	7.14E−01
*TRAF3*	34.10	35.38	0.96	3.16E−01	3.54E−01
*TNFAIP3*	100.76	104.33	0.97	4.13E−01	4.38E−01
*TICAM1*	23.76	24.47	0.97	5.30E−01	5.54E−01
*MYD88*	64.07	65.97	0.97	4.14E−01	4.38E−01
*IRAK4*	39.76	40.57	0.98	5.92E−01	6.11E−01
*MALT1*	75.70	75.80	1.00	9.69E−01	9.69E−01
*TRAF6*	38.06	36.78	1.03	3.37E−01	3.74E−01
*GADD45B*	12.90	12.05	1.07	3.43E−01	3.76E−01
*TICAM2*	8.19	7.62	1.08	2.73E−01	3.10E−01
*CARD11*	21.22	19.62	1.08	3.93E−01	4.26E−01
*LTBR*	126.02	113.31	1.11	1.41E−04	2.44E−04
*IKBKG*	6.91	6.20	1.11	2.04E−01	2.35E−01
*CSNK2B*	78.25	70.03	1.12	2.92E−04	4.80E−04
*CARD14*	12.91	11.54	1.12	1.12E−01	1.35E−01
*RELA*	82.20	73.32	1.12	5.56E−06	1.00E−05
*SYK*	111.40	98.12	1.14	1.73E−04	2.94E−04
*MAP3K7*	72.49	62.09	1.17	9.69E−07	1.86E−06
*CHUK*	44.48	37.81	1.18	4.50E−04	7.26E−04
*TNFSF11*	4.36	3.64	1.20	1.30E−01	1.56E−01
*XIAP*	187.63	156.09	1.20	8.16E−13	2.21E−12
*IL1B*	22.58	18.76	1.20	2.65E−02	3.74E−02
*TAB3*	134.33	108.39	1.24	2.64E−09	6.22E−09
*BCL2A1*	2.01	1.61	1.25	1.59E−01	1.87E−01
*CCL4*	2.74	2.06	1.33	4.03E−02	5.37E−02
*TRAF2*	40.20	29.46	1.36	8.02E−13	2.21E−12
*PARP1*	109.63	79.73	1.37	5.66E−16	1.74E−15
*ICAM1*	47.42	34.43	1.38	3.56E−07	7.28E−07
*TLR4*	51.80	37.22	1.39	1.72E−08	3.86E−08
*LYN*	48.21	33.32	1.45	2.29E−12	5.85E−12
*CSNK2A1*	138.89	92.92	1.49	5.00E−25	2.87E−24
*UBE2I*	91.18	61.00	1.49	9.53E−33	1.25E−31
*PLCG1*	199.37	118.93	1.68	8.50E−31	8.69E−30
*TRAF5*	185.37	108.42	1.71	3.65E−29	2.80E−28
*CSNK2A2*	46.72	23.14	2.02	1.14E−30	1.05E−29
*CSNK2A1P*	1.98	0.91	2.17	1.29E−12	3.39E−12
*IRAK1*	184.02	82.28	2.24	1.90E−54	8.76E−53
*BCL2L1*	144.28	64.05	2.25	1.06E−56	9.73E−55
*PLAU*	54.21	21.51	2.52	6.31E−31	7.25E−30
*CXCL2*	27.61	10.82	2.55	9.88E−20	3.95E−19
*IL8*	37.68	7.35	5.12	1.13E−25	7.46E−25

**Fig. 1:**
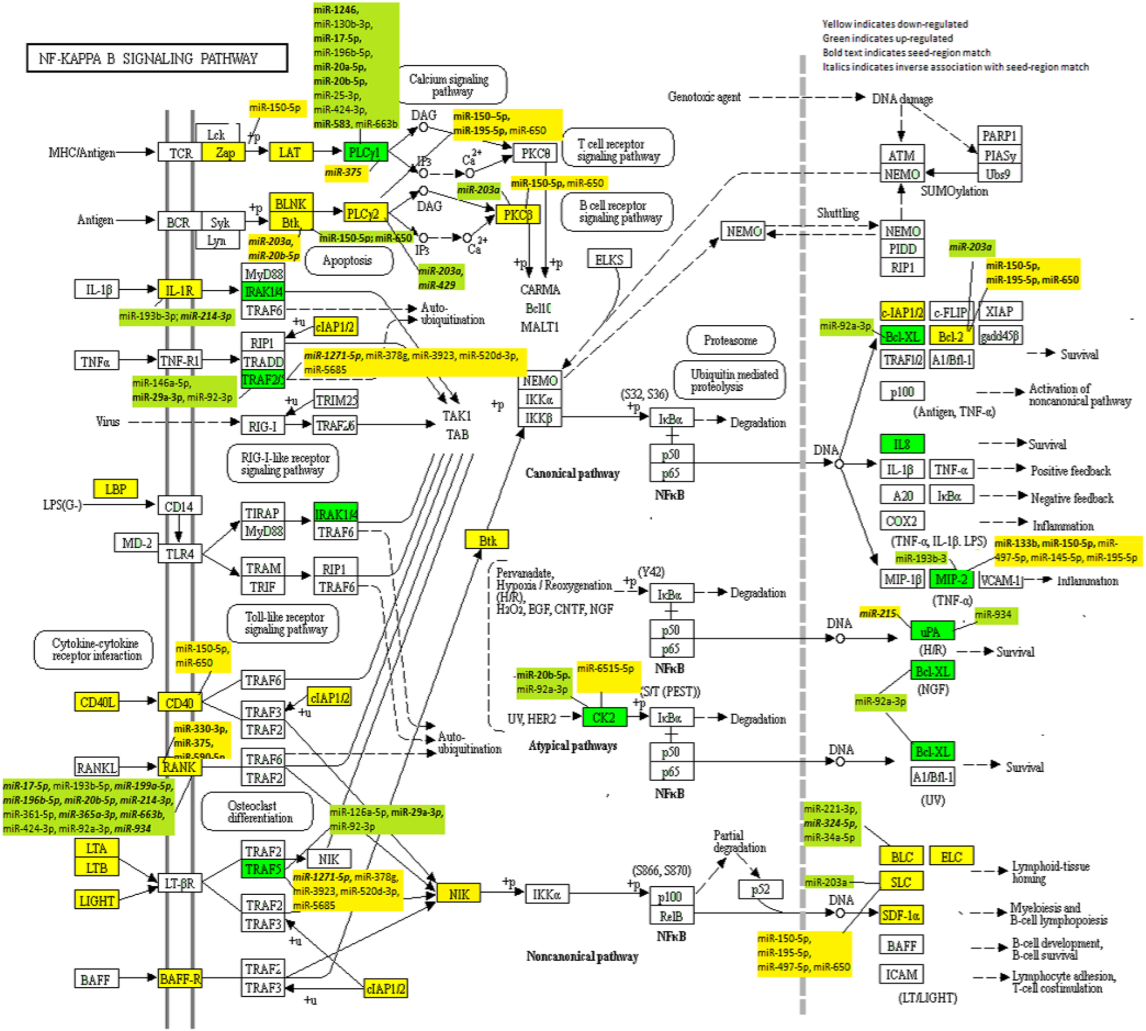
KEGG NF-κB Signalling Network with dysregulated mRNAs highlighted and their associated miRNAs included

Specific to MSS tumours, *CD14 *had a FC of 0.66 compared to a FC of 0.68 overall and *CSNK2A1* had a FC of 1.54 for MSS tumours compared to 1.49 overall (Supplemental Table 2 for MSS results). Evaluation of MSI-specific tumours showed that two additional genes were downregulated, *CARD11* and *VCAM1* (FC 0.37 and 0.59, respectively) and six genes were upregulated (*LYN* FC 1.59, *TICAM2* FC 1.64, *ICAM1* FC 1.91, *IL1B* FC 2.06, *CCL4* FC 2.72, and *PTGS2* FC 3.23) that were statistically significant (Supplemental Table 3 for MSI-specific results). Among the most downregulated genes were *CCL13* (FC 0.11), *CCL19 *(FC 0.14), *PRKCB* (FC 0.28, *TNFRSF13C* (FC0.29), and *CD40LG* (FC0.30). Seven genes were upregulated with a FC greater than 2.0 (*CSNK2A2* FC 2.02, *CSNK2A1P* FC 2.17, *IRAK1* FC 2.24, *BCL2L1* FC 2.25, *PLAU* FC 2.52,* CXCL2* FC 2.55, and *IL8 *FC 5.12). Figure 1 shows the location of these genes in the NF-κB signalling pathway.

Sixteen of the dysregulated genes were associated with 40 miRNAs (Table 3).

**Table 3:** Associations between differentially expressed genes in the NF-kB Signalling Pathway and miRNA differential expression

Gene name	Tumour mean	Normal mean	Fold change	miRNA	Tumour mean	Normal mean	Fold change	Raw *p* value	FDR *p* value
BTK	5.16	15.34	0.34	**hsa-miR-150-5p** ^**a**^	14.90	39.17	0.38	< 0.0001	0.0116
				**hsa-miR-203a**	12.52	3.70	3.38	< 0.0001	0.0116
				**hsa-miR-20b-5p**	17.65	3.30	5.35	< 0.0001	0.0116
				hsa-miR-650	4.51	16.60	0.27	< 0.0001	0.0116
CSNK2A2	46.72	23.14	2.02	**hsa-miR-20b-5p**	17.65	3.30	5.35	< 0.0001	0.0271
				hsa-miR-6515-5p	1.20	4.41	0.27	< 0.0001	0.0271
				hsa-miR-92a-3p	121.60	41.18	2.95	< 0.0001	0.0271
TRAF5	185.37	108.42	1.71	**hsa-miR-1271-5p**	1.28	2.35	0.55	< 0.0001	0.0349
				hsa-miR-146a-5p	10.73	6.93	1.55	0.0007	0.0438
				**hsa-miR-29a-3p**	110.29	51.04	2.16	0.0006	0.0438
				hsa-miR-378g	1.19	2.46	0.48	0.0007	0.0438
				hsa-miR-3923	0.29	1.66	0.18	0.0003	0.0349
				hsa-miR-520d-3p	1.74	2.76	0.63	< 0.0001	0.0349
				hsa-miR-5685	1.28	2.78	0.46	0.0003	0.0349
				hsa-miR-92a-3p	121.60	41.18	2.95	0.0003	0.0349
CD40	16.05	24.91	0.64	hsa-miR-150-5p	14.90	39.17	0.38	< 0.0001	0.0136
				hsa-miR-650	4.51	16.60	0.27	< 0.0001	0.0136
CXCL12	23.04	74.27	0.31	**hsa-miR-133b**	1.71	6.94	0.25	0.0005	0.037
				hsa-miR-145-5p	132.97	223.14	0.60	0.0003	0.037
				**hsa-miR-150-5p**	14.90	39.17	0.38	0.0005	0.037
				hsa-miR-193b-3p	9.12	5.42	1.68	< 0.0001	0.0326
				hsa-miR-195-5p	3.59	12.18	0.29	0.0002	0.0326
				hsa-miR-497-5p	1.77	7.12	0.25	0.0002	0.0326
ZAP70	7.77	18.76	0.41	hsa-miR-150-5p	14.90	39.17	0.38	< 0.0001	0.0407
IL1R1	57.71	89.40	0.65	hsa-miR-193b-3p	9.12	5.42	1.68	< 0.0001	0.0203
				**hsa-miR-214-3p**	13.24	6.13	2.16	0.0003	0.0407
PLAU	54.21	21.51	2.52	**hsa-miR-215**	49.53	77.27	0.64	0.0002	0.0407
				hsa-miR-934	4.36	0.94	4.66	< 0.0001	0.0407
PLCG1	199.37	118.93	1.68	**hsa-miR-1246**	629.21	412.81	1.52	0.0004	0.0271
				hsa-miR-130b-3p	8.74	4.89	1.79	0.0007	0.0305
				**hsa-miR-17-5p**	61.04	16.38	3.73	0.0009	0.0305
				hsa-miR-196b-5p	17.89	5.53	3.24	0.0009	0.0305
				**hsa-miR-20a-5p**	70.78	17.61	4.02	0.0009	0.0305
				**hsa-miR-20b-5p**	17.65	3.30	5.35	0.0006	0.0305
				hsa-miR-25-3p	30.05	12.78	2.35	0.0005	0.0291
				**hsa-miR-375**	20.50	54.53	0.38	0.0014	0.0407
				hsa-miR-424-3p	39.81	25.37	1.57	< 0.0001	0.0163
				**hsa-miR-583**	6.61	3.22	2.05	0.0009	0.0305
				hsa-miR-663b	65.50	32.21	2.03	0.0009	0.0305
				hsa-miR-92a-3p	121.60	41.18	2.95	< 0.0001	0.0163
CCL21	5.84	16.77	0.35	hsa-miR-150-5p	14.90	39.17	0.38	< 0.0001	0.0203
				hsa-miR-195-5p	3.59	12.18	0.29	< 0.0001	0.0203
				hsa-miR-203a	12.52	3.70	3.38	0.0002	0.0233
				hsa-miR-497-5p	1.77	7.12	0.25	0.0002	0.0233
				hsa-miR-650	4.51	16.60	0.27	0.0002	0.0233
TNFRSF11A	53.68	118.87	0.45	**hsa-miR-17-5p**	61.04	16.38	3.73	0.0021	0.0342
				hsa-miR-193b-3p	9.12	5.42	1.68	< 0.0001	0.0063
				**hsa-miR-196b-5p**	17.89	5.53	3.24	< 0.0001	0.0063
				**hsa-miR-199a-5p**	20.18	9.28	2.17	0.0007	0.0178
				**hsa-miR-20b-5p**	17.65	3.30	5.35	0.0006	0.0158
				**hsa-miR-214-3p**	13.24	6.13	2.16	0.0004	0.0125
				**hsa-miR-330-3p**	2.81	5.59	0.50	0.0015	0.0284
				hsa-miR-361-5p	11.62	6.20	1.87	0.0015	0.0284
				**hsa-miR-365a-3p**	8.43	4.33	1.94	< 0.0001	0.0063
				**hsa-miR-375**	20.50	54.53	0.38	0.0002	0.0081
				hsa-miR-424-3p	39.81	25.37	1.57	0.0004	0.0125
				**hsa-miR-590-5p**	1.78	3.04	0.59^b^	0.0029	0.0414
				**hsa-miR-663b**	65.50	32.21	2.03	0.0005	0.0151
				hsa-miR-92a-3p	121.60	41.18	2.95	0.0002	0.0081
				**hsa-miR-934**	4.36	0.94	4.66	0.0002	0.0081
PRKCB	14.65	53.12	0.28	**hsa-miR-150-5p**	14.90	39.17	0.38	< 0.0001	0.0102
				**hsa-miR-203a**	12.52	3.70	3.38	< 0.0001	0.0102
				hsa-miR-650	4.51	16.60	0.27	< 0.0001	0.0102
BCL2L1	144.28	64.05	2.25	hsa-miR-92a-3p	121.60	41.18	2.95	< 0.0001	0.0407
BCL2	24.42	62.11	0.39	**hsa-miR-150-5p**	14.90	39.17	0.38	< 0.0001	0.0203
				**hsa-miR-195-5p**	3.59	12.18	0.29	0.0003	0.0488
				**hsa-miR-203a**	12.52	3.70	3.38	< 0.0001	0.0203
				**hsa-miR-650**	4.51	16.60	0.27	< 0.0001	0.0203
CCL13	0.71	6.30	0.11	hsa-miR-221-3p	13.53	4.12	3.28	< 0.0001	0.0271
				**hsa-miR-324-5p**	5.20	2.27	2.29	0.0004	0.0452
				hsa-miR-34a-5p	25.15	12.32	2.04	0.0004	0.0452
PLCG2	16.91	52.18	0.32	**hsa-miR-150-5p**	14.90	39.17	0.38	< 0.0001	0.0163
				**hsa-miR-195-5p**	3.59	12.18	0.29	0.0002	0.0203
				**hsa-miR-203a**	12.52	3.70	3.38	0.0002	0.0203
				**hsa-miR-429**	13.33	8.29	1.61	0.0005	0.0407
				hsa-miR-650	4.51	16.60	0.27	< 0.0001	0.0163

^a^Seed-region matches in bold

^b^The beta coefficient was inverse even though both mRNA and miRNA were downregulated

Of the total 76 miRNA:mRNA associations from these 16 genes and 40 miRNAs, 38 had seed-region matches and 19 of these matches showed an inverse association between the differential expression of the miRNA and the differential expression of the mRNA. *TNFRSF11A* was associated with the greatest number of miRNAs (*N* = 15). *PLCG1* was associated with 12 miRNAs, *TRAF5* with eight miRNAs, *CXCL12* with six miRNAs, and *CCL21* and *PLCG2* each with five miRNAs. The miRNA and mRNA associations are further summarised in Table 4.

**Table 4:** Summary of NF-κB signalling pathway gene expression and miRNA associations

miRNA	Genes with seed-region match	Genes without seed-region match
miR-1246	*PLCG1*	
miR-1271-5p	***TRAF5***	
miR-130b-3p		*PLCG1*
miR-133b	*CXCL12*	
miR-145-5p		*CXCL12*
miR-146a-5p		*TRAF5*
miR-150-5p	*BTK, CXCL2, PRKCB*, *BCL2, PLCG2*,	*CD40, ZAP70, CCL21*
miR-17-5p	*PLCG1*, ***TNFRSF11A***	
miR-193b-3p		*CXCL12, TNFRSF11A, IL1R1*
miR-195-5p	*BCL2. PLCG2*	*CXCL12, CCL21*
miR-196b-5p	***TNFRSF11A***	*PLCG1*
miR-199a-5p	***TNFRSF11A***	
miR-203a	***BTK***, ***PRKCB***, ***BCL2***, ***PLCG2***	*CCL21*
miR-20a-5p	*PLCG1*	
miR-20b-5p	***BTK***, *CSNK2A2, PLCG1*, ***TNFRSF11A***	
miR-214-3p	*IL1R1*, ***TNFRSF11A***	
miR-215	***PLAU***	
miR-221-3p		*CCL13*
miR-25-3p		*PLCG1*
miR-29a-3p	*TRAF5*	
miR-324-5p	***CCL13***	
miR-330-3p	*TNFRSF11A*	
miR-34a-5p		*CCL13*
miR-361-5p		*TNFRSF11A*
miR-365a-3p	***TNFRSF11A***	
miR-375	***PLCG1***, *TNFRSF11A*	
miR-378g		*TRAF5*
miR-3923		*TRAF5*
miR-424-3p		*PLCG1, TNFRSF11A*
miR-429	***PLCG2***	
miR-497-5p		*CXCL12, CCL21*
miR-520d-3p		*TRAF5*
miR-5685		*TRAF5*
miR-583	*PLCG1*	
miR-590-5p	*TNFRSF11A*	
miR-650	*BLC2*	*BTK, CD40, CCL21, PRKCB, PLCG2*
miR-6515-5p		*CSNK2A2*
miR-663b	***TNFRSF11A***	*PLCG1*,
miR-92a-3p		*CSNK2A2, TRAF5, PLCG1, TRFRSF11A, BCL2L1*
miR-934	***TNFRSF11A***	*PLAU*

Bold text indicates inverse associations

The miRNAs with the greatest number of genes associated with these were miR-150-5p with eight genes (five with a seed-region match), miR-195-5p with four genes (two with a seed-region match), miR-203a with five genes (four with a seed-region match), miR-20b-5p with four genes (four with a seed-region match), miR-650 with six genes (one with a seed-region match), and miR-92a-3p with five genes (no seed-region matches). Interestingly, the six genes associated with miR-650 also were associated with miR-150-5p. The miRNA associations with dysregulated genes can be seen in Fig. 1.

## Discussion

The NF-κB signalling pathway is important in the carcinogenic process given its role in the regulation of genes both inside and outside of the immune system. Thus, it can potentially influence many diseases, including CRC. Our data suggest that 44.5% of NF-κB signalling pathway genes were dysregulated in colorectal carcinoma relative to expression in adjacent normal mucosa, when considering all carcinomas as well as MSS and MSI-specific carcinomas and greater levels of FC. Sixteen of these dysregulated genes were associated with 40 miRNAs. Approximately half of the 76 mRNA:miRNA associations had seed-region matches. Focusing on the genes and their associated miRNAs within the signalling pathway allows us to obtain a better understanding of how this complex pathway operates.

NF-κB activation can be involved in immune defense, especially in acute inflammatory response; it also can have be pro-inflammatory involved in pro-tumourigenic functions on the other (Hoesel and Schmid 2013). We observed dysregulated genes in both the canonical and non-canonical components of the NF-κB signalling pathway in genes suggesting both immune defense and a pro-inflammatory response. Our data showed that in the canonical pathway, the majority of genes that were downregulated were upstream of IKK and NF-κB1 while upregulated genes were mainly downstream of these central genes. Genes needed for T-cell signal transduction, *ZAP70* and *LAT* (linker for activation of T-cells) were downregulated and *PLCG1* was upregulated in the TCR arm of the pathway. Studies have shown that higher levels of *PLCG1* expression in breast tumours has been linked to metastasis (Sala et al. 2008). *BLNK*, *Btk,* *PRKCB* and *PLCG2*, critical for BCR signalling, were downregulated. Since Btk plays an important role in adaptive immunity and mutations in *PLCG2* have been associated with immune deficiency, downregulation of these genes could have a negative impact on immune response. *IL1R*, which is an antagonist to the pro-inflammatory cytokine IL1, was downregulated while the interleukin receptor kinase 1 (*IRAK1*) which could positively induce IKK was upregulated. *TRAF5*, downstream from TNF-R1, which is a positive regulator of IKK and NF-κB, was upregulated. These alterations in gene expression could influence immune response and promote inflammation by downregulation of genes that enable immune response and upregulation of pro-inflammatory genes.

Interestingly, *NEMO* (IKBKγ) (FC 1.11), *IKBKB* (FC 0.94), *NFκB1* (FC 0.92) and *RELA* (FC 1.12) were only slightly changed in carcinoma tissue compared to normal mucosa. Given that NF-κB is a transcription factor, it is possible that its expression is more tightly controlled through feedback loops. However, once the system is activated by dysregulated upstream genes, an active NF-κB can stimulate expression of downstream genes. Downstream of the IKK and NF-κB, several major genes were upregulated, including *BCL21*, *IL8* (*CXCL8*) and *CXCL2* (MIP-2 in pathway). *BLC2* (which was downregulated) and *BCL2L1* are anti-apoptotic; *IL8 *and *CXCL2* are chemokines that are pro-inflammatory and enhance the proliferation and survival of cancer cells (Waugh and Wilson 2008).

Activators of the non-canonical NF-κB signalling pathway were for the most part downregulated. These included *CD40LG*, *CD40*, *RANK* (TNFSF11A), *LTA* (*TNFSF1*) *LTB* (*TNFSF3*), *LIGHT* (*TNFSF14*), *BAFF-R* (B-cell activating receptor coded by *TNFSF13*) which are all part of the TNF super family and key regulators of immune response. Likewise NIK (MAP3K14) was also downregulated and is the major intermediary molecule to IKK and NFKB2 activation. Three downstream genes of *NIK*, *CCL13*, *CCL19* and *CCL21*, were downregulated; these genes are part of a family of CC cytokines and play a role in inflammatory response and normal lymphocyte recirculating.

Several miRNAs have been linked to genes within the NF-κB signalling pathway (Ma et al. 2011). Many of the miRNAs previously studied that have been targeted with NF-κB signalling, such as miR-21, have been linked to both immune response and inflammation (Mima et al. 2016; Schetter et al. 2009), have been shown to be mediated by NF-κB in response to oxidative stress (Wei et al. 2014), or have been associated with specific cancers such as gastric cancer (Sha et al. 2014). In this study, we only evaluated miRNAs with genes that had a more meaningful level of change in expression in carcinoma relative to normal mucosa. Likewise, our analysis focused on CRC tissue and findings from other tissue sources may not be relevant for CRC. We did not observe miR-21 to be associated with any of the dysregulated genes evaluated after adjustment for multiple comparisons. Other miRNAs associated with inflammation and immune response (Contreras and Rao 2012) that have been linked to the NF-κB pathway for which we did not observe an association in this study were: miR-181b, previously associated with *CYLD*, which has a negative regulator effect on NF-κB; miR-301a, which has previously been shown to indirectly activate NF-κB via downregulation of NF-κB repressing factor (NKBF); miR-155, previously associated with BCR-related genes and the expression of *IL8* (Ma et al. 2011). It also has been suggested that miR-15a, miR-16 and miR-223, which are important in innate immune cells, may be important in the non-canonical NF-κB pathway (Li et al. 2010); we did not observe any meaningful associations between these miRNAs and pathway genes. However, miR-146a which has been shown to be associated with *IRAK*1 and *TRAF6* and upregulated by IL-1β and TNF (Hill et al. 2015) was upregulated in our data when *TRAF5* (tumour necrosis factor receptor-associated 5) also was significantly upregulated. MiR-199 has previously been associated with *IKKB* (Contreras and Rao 2012); we observed an association with a seed-region match between *TNFRSF11A* (RANK) and miR-199a-5p. Likewise, miR-221 has been reported as being associated with TNFα (Contreras and Rao 2012); miR-221-3p was associated with *CCL13* in the non-canonical NF-κB pathway in our data. *TRAF5* has been associated with miR-26b in melanoma cells (Li et al. 2016a, b). While we observed eight miRNAs associated with *TRAF5*, we did not observe an association with miR-26b in CRC tissue. MiR-503 has been shown to target *RANK* in osteoclastogenesis (Chen et al. 2014). *TNFRSF11A* (i.e. *RANK*) was associated with 15 miRNAs in our data, although not associated with miR-503. Other miRNAs, including let-7, miR-9, miR-143 and miR-224 that have been shown to be transcriptional targets of NF-κB (Hoesel and Schmid 2013) were not associated with genes evaluated in our colorectal data. Lack of confirmation of previous findings could be the result of our larger study with more power. Additionally since many studies have examined few miRNAs whereas we examined hundreds, differences in observed associations could be from our adjustment for multiple comparisons.

We identified several miRNAs that appear to be important with dysregulated genes in the NF-κB signalling pathway; many of these miRNAs were associated with multiple genes that further suggest their importance in the pathway in CRC. MiR-150-5p was associated with eight genes and had seed-region matches with five of these genes. MiR-150 has previously been associated with immune response (Tsitsiou and Lindsay 2009), with reducing inflammatory cytokine production (Sang et al. 2016), and was one of 25 miRNAs that were important in distinguishing rectal carcinomas from normal rectal mucosa in our data (Pellatt et al. 2016). Interestingly, six of these eight miRNAs associated with miR-150-5p were also associated with miR-650. Both miR-203a and miR-20b-5p had seed-region matches with four genes in our data while miR-92a-3p was associated with five genes, although none with a seed-region match. Both miR-20b-5p and miR-92a-3p are members of the miR-17-92 cluster, which has been associated with CRC and *MYC* expression and Wnt signalling (Li et al. 2014, 2016; Ma et al. 2016).

Several pathway genes were associated with numerous miRNAs. Some of these associations had seed-region matches which implied a greater likelihood of binding that could alter expression. Seed-region matches for pairs that were inversely associated in that either the mRNA or miRNA was upregulated while the other was downregulated, suggest binding that has a greater likelihood of directly altering function. However, miRNA:mRNA associations that had the same directionality in terms of FC, suggest an indirect effect or a feedback loop that results in both miRNA and mRNA being either up or downregulated. *TRAF5* was associated with eight miRNAs (direct seed-region matches with two miRNAs); *PLCG1* was associated with 12 miRNAs (six with seed-region matches and one, miR-375, had an inverse association suggesting a direct effect on each other); *TNFRSF11A* was associated with 15 miRNAs, 11 of which had a seed-region match (nine of these seed-region matches suggest direct binding of the mRNA to the miRNA). TRAFs were originally shown to be a signal-transducing molecule for *TNFR *and *IL1R* (Tada et al. 2001). *PLCG1* is generally activated by growth factor receptors such as vascular endothelial growth factor receptors (VEGFRs), insulin-like growth factor 1 receptor (IGF-1R) and FGFRs and has been linked to metastatic tumour progression (Lattanzio et al. 2013). *TNFRSF11A* or *RANK* expression in breast tumours has been found predominately in those tumours with a high grade and proliferation index (Sigl et al. 2016); it has also received attention as a possible therapeutic agent (Sigl et al. 2016; Gonzalez-Suarez and Sanz-Moreno 2016; Jimi et al. 2013).

There are several considerations when interpreting results from this study. Although our sample size is small, it is one of the largest available containing samples with paired carcinoma and normal mucosa data. While normal colonic mucosa may not be truly “normal”, it is the closest normal mucosa that can be used for a matched paired analysis. Furthermore, normal colonic mucosa was taken from the same colonic site as the tumour to prevent differences in expression from being the result of tumour location. We focused only on those genes that were statistically significant and also had a FC of > 1.5 or < 0.67. Using these criteria, we did not examine all statistically significant genes that were differentially expressed and miRNAs. A biologically important FC is not well defined, and using set values for further follow-up we could have missed genes associated with miRNAs. We exclusively used the KEGG pathway database to identify signalling pathway genes. Genes not identified in KEGG but associated with the NF-κB signalling pathway may importantly influence miRNA expression, but were not included in this analysis. When evaluating miRNA with mRNAs, we could have missed important associations since miRNAs may have their impact post-transcriptionally and we were only able to evaluate gene expression. However, much of the current information on miRNA target genes comes from gene expression data and associations observed may have important biological meaning, but must be acknowledged as being incomplete (Chou et al. 2016; Slattery et al. 2017). Thus, we encourage others to both replicate these findings and validate results in targeted laboratory experiments.

In conclusion, the NF-κB signalling pathway is a complex pathway that is important in the carcinogenic process of CRC. The alterations in gene expression observed in signalling pathway genes could influence immune response and promote inflammation through its downregulation of genes that have a positive role of immune response and upregulation of genes that are pro-inflammatory. Through examining of the entire pathway, we believe that we have obtained a more thorough understanding of those components of the pathway most important for CRC. It is important to have a comprehensive understanding of the complex associations that exist between mRNAs and miRNAs that co-regulate the pathway in order to accurately identify therapeutic targets, such as *RANK*.

## Electronic supplementary material

Below is the link to the electronic supplementary material.

Supplementary material 1 (DOCX 49 KB)
