# Supplementary material for: The NF-κB signalling pathway in colorectal cancer: associations between dysregulated gene and miRNA expression
Source: J Cancer Res Clin Oncol. 2017 Nov 29;144(2):269–83. doi: 10.1007/s00432-017-2548-6 (PMC5794831; doi:10.1007/s00432-017-2548-6)
Supplement: Supplementary file 1 — Supplementary material 1 (DOCX 49 KB) [file 432_2017_2548_MOESM1_ESM.docx]

| Supplemental Table 1. List of KEGG NF Kappa B Signaling Pathway Genes | | | | | |  |
| --- | --- | --- | --- | --- | --- | --- |
| KEGG Gene ID | Gene Name | Description |  |  |  |  |
| 3932 | *LCK* | LCK proto-oncogene, Src family tyrosine kinase [KO:K05856] [EC:2.7.10.2] | | | | |
| 7535 | *ZAP70* | zeta chain of T-cell receptor associated protein kinase 70 [KO:K07360] [EC:2.7.10.2] | | | | |
| 27040 | *LAT* | linker for activation of T-cells [KO:K07362] | | | |  |
| 5335 | *PLCG1* | phospholipase C gamma 1 [KO:K01116] [EC:3.1.4.11] | | | | |
| 5588 | *PRKCQ* | protein kinase C theta [KO:K18052] [EC:2.7.11.13] | | | |  |
| 102723407 | *IGH* | putative V-set and immunoglobulin domain-containing-like protein IGHV4OR15-8 [KO:K06856] | | | | |
| 6850 | *SYK* | spleen associated tyrosine kinase [KO:K05855] [EC:2.7.10.2] | | | | |
| 4067 | *LYN* | LYN proto-oncogene, Src family tyrosine kinase [KO:K05854] [EC:2.7.10.2] | | | | |
| 29760 | *BLNK* | B-cell linker [KO:K07371] | |  |  |  |
| 695 | *BTK* | Bruton tyrosine kinase [KO:K07370] [EC:2.7.10.2] | | | |  |
| 5336 | *PLCG2* | phospholipase C gamma 2 [KO:K05859] [EC:3.1.4.11] | | | | |
| 5579 | *PRKCB* | protein kinase C beta [KO:K19662] [EC:2.7.11.13] | | | |  |
| 29775 | *CARD10* | caspase recruitment domain family member 10 [KO:K20912] | | | | |
| 84433 | *CARD11* | caspase recruitment domain family member 11 [KO:K07367] | | | | |
| 79092 | *CARD14* | caspase recruitment domain family member 14 [KO:K20913] | | | | |
| 8915 | *BCL10* | B-cell CLL/lymphoma 10 [KO:K07368] | | |  |  |
| 10892 | *MALT1* | MALT1 paracaspase [KO:K07369] [EC:3.4.22.-] | | | |  |
| 3553 | *IL1B* | interleukin 1 beta [KO:K04519] | | |  |  |
| 3554 | *IL1R1* | interleukin 1 receptor type 1 [KO:K04386] | | | |  |
| 4615 | *MYD88* | myeloid differentiation primary response 88 [KO:K04729] | | | | |
| 3654 | *IRAK1* | interleukin 1 receptor associated kinase 1 [KO:K04730] [EC:2.7.11.1] | | | | |
| 51135 | *IRAK4* | interleukin 1 receptor associated kinase 4 [KO:K04733] [EC:2.7.11.1] | | | | |
| 7189 | *TRAF6* | TNF receptor associated factor 6 [KO:K03175] [EC:2.3.2.27] | | | | |
| 7124 | *TNF* | tumor necrosis factor [KO:K03156] | | |  |  |
| 7132 | *TNFRSF1A* | TNF receptor superfamily member 1A [KO:K03158] | | | |  |
| 8737 | *RIPK1* | receptor interacting serine/threonine kinase 1 [KO:K02861] [EC:2.7.11.1] | | | | |
| 8717 | *TRADD* | TNFRSF1A associated via death domain [KO:K03171] | | | | |
| 7186 | *TRAF2* | TNF receptor associated factor 2 [KO:K03173] [EC:2.3.2.27] | | | | |
| 7188 | *TRAF5* | TNF receptor associated factor 5 [KO:K09849] | | | |  |
| 329 | *BIRC2* | baculoviral IAP repeat containing 2 [KO:K16060] | | | |  |
| 330 | *BIRC3* | baculoviral IAP repeat containing 3 [KO:K16060] | | | |  |
| 23586 | *DDX58* | DExD/H-box helicase 58 [KO:K12646] [EC:3.6.3.14] | | | |  |
| 7706 | *TRIM25* | tripartite motif containing 25 [KO:K10652] [EC:2.3.2.27] | | | | |
| 3929 | *LBP* | lipopolysaccharide binding protein [KO:K05399] | | | |  |
| 929 | *CD14* | CD14 molecule [KO:K04391] | |  |  |  |
| 7099 | *TLR4* | toll like receptor 4 [KO:K10160] | | |  |  |
| 23643 | *LY96* | lymphocyte antigen 96 [KO:K05400] | | |  |  |
| 114609 | *TIRAP* | TIR domain containing adaptor protein [KO:K05403] | | | |  |
| 353376 | *TICAM2* | toll like receptor adaptor molecule 2 [KO:K05409] | | | |  |
| 148022 | *TICAM1* | toll like receptor adaptor molecule 1 [KO:K05842] | | | |  |
| 959 | *CD40LG* | CD40 ligand [KO:K03161] | |  |  |  |
| 958 | *CD40* | CD40 molecule [KO:K03160] | |  |  |  |
| 7187 | *TRAF3* | TNF receptor associated factor 3 [KO:K03174] | | | |  |
| 8600 | *TNFSF11* | TNF superfamily member 11 [KO:K05473] | | | |  |
| 8792 | *TNFRSF11A* | TNF receptor superfamily member 11a [KO:K05147] | | | |  |
| 4049 | *LTA* | lymphotoxin alpha [KO:K05468] | | |  |  |
| 4050 | *LTB* | lymphotoxin beta [KO:K03157] | | |  |  |
| 8740 | *TNFSF14* | TNF superfamily member 14 [KO:K05477] | | | |  |
| 4055 | *LTBR* | lymphotoxin beta receptor [KO:K03159] | | |  |  |
| 9020 | *MAP3K14* | mitogen-activated protein kinase kinase kinase 14 [KO:K04466] [EC:2.7.11.25] | | | | |
| 6885 | *MAP3K7* | mitogen-activated protein kinase kinase kinase 7 [KO:K04427] [EC:2.7.11.25] | | | | |
| 10454 | *TAB1* | TGF-beta activated kinase 1 (MAP3K7) binding protein 1 [KO:K04403] | | | | |
| 23118 | *TAB2* | TGF-beta activated kinase 1/MAP3K7 binding protein 2 [KO:K04404] | | | | |
| 257397 | *TAB3* | TGF-beta activated kinase 1 and MAP3K7 binding protein 3 [KO:K12793] | | | | |
| 10673 | *TNFSF13B* | TNF superfamily member 13b [KO:K05476] | | | |  |
| 115650 | *TNFRSF13C* | TNF receptor superfamily member 13C [KO:K05151] | | | | |
| 8517 | *IKBKG* | inhibitor of nuclear factor kappa B kinase subunit gamma [KO:K07210] | | | | |
| 1147 | *CHUK* | conserved helix-loop-helix ubiquitous kinase [KO:K04467] [EC:2.7.11.10] | | | | |
| 3551 | *IKBKB* | inhibitor of nuclear factor kappa B kinase subunit beta [KO:K07209] [EC:2.7.11.10] | | | | |
| 51588 | *PIAS4* | protein inhibitor of activated STAT 4 [KO:K16065] | | | |  |
| 7329 | *UBE2I* | ubiquitin conjugating enzyme E2 I [KO:K10577] | | | |  |
| 472 | *ATM* | ATM serine/threonine kinase [KO:K04728] [EC:2.7.11.1] | | | | |
| 55367 | *PIDD1* | p53-induced death domain protein 1 [KO:K10130] | | | |  |
| 23085 | *ERC1* | ELKS/RAB6-interacting/CAST family member 1 [KO:K16072] | | | | |
| 4792 | *NFKBIA* | NFKB inhibitor alpha [KO:K04734] | | |  |  |
| 4790 | *NFKB1* | nuclear factor kappa B subunit 1 [KO:K02580] | | | |  |
| 5970 | *RELA* | RELA proto-oncogene, NF-kB subunit [KO:K04735] | | | |  |
| 8837 | *CFLAR* | CASP8 and FADD like apoptosis regulator [KO:K04724] | | | | |
| 331 | *XIAP* | X-linked inhibitor of apoptosis [KO:K04725] [EC:2.3.2.27] | | | | |
| 598 | *BCL2L1* | BCL2 like 1 [KO:K04570] | |  |  |  |
| 596 | *BCL2* | BCL2, apoptosis regulator [KO:K02161] | | |  |  |
| 7185 | *TRAF1* | TNF receptor associated factor 1 [KO:K03172] | | | |  |
| 597 | *BCL2A1* | BCL2 related protein A1 [KO:K02162] | | |  |  |
| 4791 | *NFKB2* | nuclear factor kappa B subunit 2 [KO:K04469] | | | |  |
| 3576 | *CXCL8* | C-X-C motif chemokine ligand 8 [KO:K10030] | | | |  |
| 7128 | *TNFAIP3* | TNF alpha induced protein 3 [KO:K11859] [EC:3.4.19.12] | | | | |
| 5743 | *PTGS2* | prostaglandin-endoperoxide synthase 2 [KO:K11987] [EC:1.14.99.1] | | | | |
| 6351 | *CCL4* | C-C motif chemokine ligand 4 [KO:K12964] | | | |  |
| 9560 | *CCL4L2* | C-C motif chemokine ligand 4 like 2 [KO:K12964] | | | |  |
| 388372 | *CCL4L1* | C-C motif chemokine ligand 4 like 1 [KO:K12964] | | | |  |
| 7412 | *VCAM1* | vascular cell adhesion molecule 1 [KO:K06527] | | | |  |
| 5328 | *PLAU* | plasminogen activator, urokinase [KO:K01348] [EC:3.4.21.73] | | | | |
| 1457 | *CSNK2A1* | casein kinase 2 alpha 1 [KO:K03097] [EC:2.7.11.1] | | | |  |
| 1459 | *CSNK2A2* | casein kinase 2 alpha 2 [KO:K03097] [EC:2.7.11.1] | | | |  |
| 283106 | *CSNK2A3* | casein kinase 2 alpha 3 [KO:K03097] [EC:2.7.11.1] | | | |  |
| 1460 | *CSNK2B* | casein kinase 2 beta [KO:K03115] | | |  |  |
| 5971 | *RELB* | RELB proto-oncogene, NF-kB subunit [KO:K09253] | | | |  |
| 6357 | *CCL13* | C-C motif chemokine ligand 13 [KO:K16595] | | | |  |
| 6363 | *CCL19* | C-C motif chemokine ligand 19 [KO:K05512] | | | |  |
| 6366 | *CCL21* | C-C motif chemokine ligand 21 [KO:K16062] | | | |  |
| 6387 | *CXCL12* | C-X-C motif chemokine ligand 12 [KO:K10031] | | | |  |
| 3383 | *ICAM1* | intercellular adhesion molecule 1 [KO:K06490] | | | |  |
| 142 | *PARP1* | poly(ADP-ribose) polymerase 1 [KO:K10798] [EC:2.4.2.30] | | | | |
| 2920 | *CXCL2* | C-X-C motif chemokine ligand 2 [KO:K05505] | | | |  |
| 4616 | *GADD45B* | growth arrest and DNA damage inducible beta [KO:K04402] | | | | |

| Supplemental Table 2. Associations between differential expression of NF-κB-Signaling Pathway Genes in MSS Tumors | | | | | |
| --- | --- | --- | --- | --- | --- |
| Gene Name | Tumor Mean | Normal Mean | Fold Change | P-Value | Adjusted P-Value |
| *CCL13* | 0.59 | 5.83 | 0.10 | 2.22E-21 | 9.74E-21 |
| *CCL19* | 0.95 | 6.77 | 0.14 | 1.39E-21 | 6.39E-21 |
| *TNFRSF13C* | 1.44 | 5.33 | 0.27 | 2.99E-16 | 1.14E-15 |
| *PRKCB* | 13.53 | 49.63 | 0.27 | 3.87E-37 | 8.91E-36 |
| *CXCL12* | 22.53 | 74.92 | 0.30 | 7.40E-47 | 2.27E-45 |
| *PLCG2* | 16.24 | 50.46 | 0.32 | 1.83E-32 | 2.41E-31 |
| *CCL21* | 5.34 | 16.52 | 0.32 | 5.28E-16 | 1.94E-15 |
| *CD40LG* | 1.02 | 3.13 | 0.32 | 5.12E-13 | 1.52E-12 |
| *BTK* | 5.03 | 14.84 | 0.34 | 1.23E-21 | 5.93E-21 |
| *TNFRSF11A* | 44.01 | 121.83 | 0.36 | 1.43E-29 | 1.28E-28 |
| *BCL2* | 22.74 | 61.38 | 0.37 | 1.93E-36 | 3.55E-35 |
| *ZAP70* | 7.35 | 18.02 | 0.41 | 6.30E-19 | 2.63E-18 |
| *BLNK* | 19.06 | 45.39 | 0.42 | 1.70E-25 | 1.11E-24 |
| *LTB* | 3.11 | 7.18 | 0.43 | 6.56E-11 | 1.77E-10 |
| *LTA* | 0.61 | 1.17 | 0.52 | 1.58E-03 | 2.55E-03 |
| *BIRC3* | 71.93 | 113.35 | 0.63 | 7.98E-15 | 2.82E-14 |
| *LBP* | 0.23 | 0.35 | 0.65 | 1.46E-01 | 1.77E-01 |
| *LAT* | 7.70 | 11.93 | 0.65 | 2.54E-08 | 5.69E-08 |
| *MAP3K14* | 33.49 | 51.65 | 0.65 | 1.30E-23 | 7.96E-23 |
| *IL1R1* | 57.04 | 86.97 | 0.66 | 3.76E-14 | 1.23E-13 |
| *CD14* | 9.45 | 14.25 | 0.66 | 1.12E-07 | 2.41E-07 |
| *TNFSF14* | 6.30 | 9.38 | 0.67 | 5.09E-05 | 9.19E-05 |
| *CD40* | 15.63 | 23.00 | 0.68 | 4.51E-06 | 8.82E-06 |
| *CFLAR* | 202.93 | 293.76 | 0.69 | 6.56E-28 | 4.64E-27 |
| *RIPK1* | 49.00 | 69.33 | 0.71 | 1.71E-16 | 6.83E-16 |
| *TNFRSF1A* | 87.61 | 118.88 | 0.74 | 7.39E-23 | 4.00E-22 |
| *BCL10* | 41.21 | 55.61 | 0.74 | 9.91E-10 | 2.34E-09 |
| *NFKBIA* | 58.25 | 76.46 | 0.76 | 5.31E-08 | 1.16E-07 |
| *LCK* | 7.72 | 10.11 | 0.76 | 7.22E-03 | 1.07E-02 |
| *ATM* | 234.61 | 305.26 | 0.77 | 7.79E-14 | 2.47E-13 |
| *PTGS2* | 13.18 | 17.00 | 0.78 | 1.96E-02 | 2.77E-02 |
| *TIRAP* | 21.36 | 27.18 | 0.79 | 1.49E-06 | 2.97E-06 |
| *LY96* | 0.92 | 1.15 | 0.80 | 2.75E-01 | 3.09E-01 |
| *PRKCQ* | 7.01 | 8.74 | 0.80 | 1.77E-02 | 2.54E-02 |
| *PIAS4* | 29.06 | 35.52 | 0.82 | 5.05E-06 | 9.69E-06 |
| *BIRC2* | 78.61 | 95.62 | 0.82 | 1.04E-09 | 2.40E-09 |
| *TNFSF13B* | 4.30 | 5.03 | 0.85 | 1.06E-01 | 1.30E-01 |
| *RELB* | 24.69 | 28.67 | 0.86 | 3.26E-03 | 5.18E-03 |
| *TRADD* | 25.47 | 29.48 | 0.86 | 4.22E-03 | 6.47E-03 |
| *TAB1* | 28.72 | 33.07 | 0.87 | 6.07E-04 | 9.97E-04 |
| *TAB2* | 126.20 | 142.60 | 0.89 | 1.54E-05 | 2.83E-05 |
| *VCAM1* | 13.62 | 15.34 | 0.89 | 1.85E-01 | 2.18E-01 |
| *NFKB1* | 74.35 | 83.07 | 0.90 | 5.04E-04 | 8.42E-04 |
| *NFKB2* | 43.48 | 48.47 | 0.90 | 2.37E-02 | 3.31E-02 |
| *TRIM25* | 135.62 | 151.14 | 0.90 | 1.20E-04 | 2.08E-04 |
| *DDX58* | 42.90 | 47.38 | 0.91 | 8.51E-02 | 1.07E-01 |
| *CARD10* | 72.58 | 79.57 | 0.91 | 3.97E-02 | 5.29E-02 |
| *ERC1* | 143.17 | 155.61 | 0.92 | 1.03E-02 | 1.50E-02 |
| *TRAF1* | 46.94 | 50.77 | 0.92 | 1.02E-01 | 1.26E-01 |
| *PIDD* | 29.34 | 31.18 | 0.94 | 2.03E-01 | 2.37E-01 |
| *IKBKB* | 139.63 | 146.41 | 0.95 | 1.80E-01 | 2.16E-01 |
| *TRAF3* | 33.36 | 34.87 | 0.96 | 2.60E-01 | 2.99E-01 |
| *TICAM1* | 24.31 | 25.13 | 0.97 | 4.99E-01 | 5.28E-01 |
| *MYD88* | 63.31 | 65.24 | 0.97 | 4.47E-01 | 4.78E-01 |
| *TNF* | 1.94 | 2.00 | 0.97 | 8.43E-01 | 8.52E-01 |
| *TNFAIP3* | 102.52 | 104.69 | 0.98 | 6.53E-01 | 6.68E-01 |
| *IRAK4* | 39.38 | 40.12 | 0.98 | 6.37E-01 | 6.66E-01 |
| *MALT1* | 71.96 | 73.21 | 0.98 | 6.51E-01 | 6.68E-01 |
| *TICAM2* | 7.47 | 7.52 | 0.99 | 9.21E-01 | 9.21E-01 |
| *TRAF6* | 37.66 | 36.47 | 1.03 | 4.13E-01 | 4.52E-01 |
| *LTBR* | 123.43 | 114.10 | 1.08 | 5.55E-03 | 8.37E-03 |
| *IL1B* | 20.45 | 18.71 | 1.09 | 3.09E-01 | 3.43E-01 |
| *IKBKG* | 7.43 | 6.74 | 1.10 | 2.64E-01 | 3.00E-01 |
| *CCL4* | 2.49 | 2.22 | 1.12 | 4.43E-01 | 4.78E-01 |
| *RELA* | 83.08 | 74.02 | 1.12 | 1.40E-05 | 2.63E-05 |
| *CSNK2B* | 79.09 | 69.69 | 1.13 | 1.78E-04 | 3.04E-04 |
| *CARD14* | 13.30 | 11.66 | 1.14 | 7.80E-02 | 9.98E-02 |
| *GADD45B* | 13.22 | 11.56 | 1.14 | 7.81E-02 | 9.98E-02 |
| *CHUK* | 42.41 | 36.69 | 1.16 | 4.17E-03 | 6.47E-03 |
| *MAP3K7* | 72.57 | 61.36 | 1.18 | 1.06E-06 | 2.16E-06 |
| *SYK* | 114.91 | 95.25 | 1.21 | 2.08E-07 | 4.34E-07 |
| *XIAP* | 190.20 | 155.47 | 1.22 | 8.83E-13 | 2.54E-12 |
| *CARD11* | 22.92 | 18.40 | 1.25 | 2.72E-02 | 3.74E-02 |
| *TAB3* | 137.42 | 106.87 | 1.29 | 9.76E-11 | 2.49E-10 |
| *ICAM1* | 44.30 | 33.95 | 1.30 | 5.19E-05 | 9.19E-05 |
| *TNFSF11* | 4.55 | 3.46 | 1.32 | 3.02E-02 | 4.08E-02 |
| *PARP1* | 105.04 | 77.19 | 1.36 | 8.30E-14 | 2.54E-13 |
| *BCL2A1* | 1.80 | 1.31 | 1.37 | 6.63E-02 | 8.71E-02 |
| *TRAF2* | 40.71 | 29.42 | 1.38 | 6.02E-12 | 1.68E-11 |
| *LYN* | 44.75 | 31.63 | 1.41 | 2.21E-10 | 5.50E-10 |
| *TLR4* | 52.60 | 35.46 | 1.48 | 6.49E-10 | 1.57E-09 |
| *UBE2I* | 90.87 | 60.71 | 1.50 | 3.93E-30 | 4.02E-29 |
| *CSNK2A1* | 140.42 | 91.21 | 1.54 | 2.78E-23 | 1.60E-22 |
| *PLCG1* | 212.82 | 119.40 | 1.78 | 9.28E-33 | 1.42E-31 |
| *TRAF5* | 192.13 | 106.10 | 1.81 | 2.82E-29 | 2.16E-28 |
| *CSNK2A1P* | 2.34 | 1.15 | 2.04 | 7.23E-11 | 1.90E-10 |
| *CSNK2A2* | 49.08 | 22.30 | 2.20 | 4.24E-32 | 4.87E-31 |
| *CXCL2* | 24.08 | 10.81 | 2.23 | 1.46E-14 | 4.98E-14 |
| *IRAK1* | 191.20 | 82.21 | 2.33 | 2.63E-51 | 1.21E-49 |
| *BCL2L1* | 151.06 | 63.73 | 2.37 | 4.40E-54 | 4.05E-52 |
| *PLAU* | 53.61 | 20.65 | 2.60 | 1.54E-29 | 1.28E-28 |
| *IL8* | 31.07 | 6.46 | 4.81 | 5.80E-22 | 2.96E-21 |

| Supplemental Table 3. Associations between differential expression of NF-κB-Signaling Pathway Genes in MSI Tumors | | | | | |
| --- | --- | --- | --- | --- | --- |
| Gene Name | Tumor Mean | Normal Mean | Fold Change | P-Value | Adjusted P-Value |
| *LBP* | 0.07 | 0.83 | 0.09 | 4.88E-02 | 9.76E-02 |
| *CD40LG* | 0.66 | 4.28 | 0.15 | 3.02E-07 | 2.78E-05 |
| *CCL13* | 1.51 | 9.33 | 0.16 | 8.72E-05 | 6.17E-04 |
| *CCL19* | 1.91 | 9.83 | 0.19 | 2.35E-02 | 5.15E-02 |
| *BTK* | 5.77 | 18.77 | 0.31 | 4.19E-05 | 6.17E-04 |
| *PRKCB* | 25.53 | 79.68 | 0.32 | 5.68E-06 | 1.45E-04 |
| *PLCG2* | 21.56 | 65.14 | 0.33 | 6.32E-06 | 1.45E-04 |
| *LTA* | 0.59 | 1.66 | 0.35 | 6.43E-03 | 1.74E-02 |
| *CXCL12* | 24.43 | 67.95 | 0.36 | 7.68E-05 | 6.17E-04 |
| *CARD11* | 10.34 | 27.75 | 0.37 | 6.21E-05 | 6.17E-04 |
| *TNFRSF13C* | 4.34 | 11.47 | 0.38 | 7.89E-03 | 2.07E-02 |
| *LTB* | 3.08 | 7.79 | 0.40 | 1.91E-03 | 7.05E-03 |
| *LY96* | 0.88 | 1.99 | 0.44 | 9.06E-02 | 1.67E-01 |
| *ZAP70* | 11.02 | 24.54 | 0.45 | 3.25E-04 | 1.87E-03 |
| *TNFSF14* | 6.27 | 12.65 | 0.50 | 2.75E-03 | 9.37E-03 |
| *CCL21* | 9.42 | 18.63 | 0.51 | 2.83E-02 | 5.92E-02 |
| *CD40* | 20.33 | 39.87 | 0.51 | 1.79E-03 | 6.87E-03 |
| *BCL2* | 36.13 | 68.16 | 0.53 | 2.78E-05 | 5.11E-04 |
| *BLNK* | 34.44 | 62.71 | 0.55 | 1.20E-03 | 5.01E-03 |
| *IL1R1* | 59.97 | 104.13 | 0.58 | 3.68E-04 | 1.99E-03 |
| *VCAM1* | 10.31 | 17.35 | 0.59 | 8.92E-03 | 2.22E-02 |
| *LAT* | 8.20 | 12.97 | 0.63 | 1.02E-02 | 2.41E-02 |
| *TNFSF11* | 2.90 | 4.53 | 0.64 | 1.91E-01 | 3.02E-01 |
| *MAP3K14* | 35.95 | 51.93 | 0.69 | 4.17E-03 | 1.32E-02 |
| *GADD45B* | 10.44 | 14.69 | 0.71 | 9.38E-02 | 1.69E-01 |
| *SYK* | 83.95 | 117.01 | 0.72 | 8.53E-05 | 6.17E-04 |
| *RIPK1* | 52.25 | 72.08 | 0.72 | 2.34E-04 | 1.53E-03 |
| *ATM* | 269.39 | 371.32 | 0.73 | 6.90E-04 | 3.34E-03 |
| *TRAF1* | 45.11 | 59.84 | 0.75 | 3.86E-02 | 7.90E-02 |
| *TNF* | 2.01 | 2.65 | 0.76 | 4.33E-01 | 5.95E-01 |
| *CFLAR* | 210.11 | 271.49 | 0.77 | 5.20E-03 | 1.45E-02 |
| *PIAS4* | 31.62 | 38.17 | 0.83 | 5.51E-02 | 1.08E-01 |
| *BIRC3* | 153.28 | 183.89 | 0.83 | 2.05E-01 | 3.14E-01 |
| *TAB2* | 145.87 | 174.26 | 0.84 | 1.47E-02 | 3.30E-02 |
| *CD14* | 10.50 | 12.53 | 0.84 | 3.73E-01 | 5.28E-01 |
| *IKBKB* | 120.32 | 143.21 | 0.84 | 2.52E-02 | 5.38E-02 |
| *ERC1* | 125.77 | 146.45 | 0.86 | 6.18E-02 | 1.16E-01 |
| *TNFAIP3* | 89.55 | 103.65 | 0.86 | 1.26E-01 | 2.15E-01 |
| *PIDD* | 26.78 | 30.88 | 0.87 | 3.12E-01 | 4.56E-01 |
| *RELB* | 26.30 | 29.93 | 0.88 | 2.05E-01 | 3.14E-01 |
| *TRADD* | 22.94 | 25.98 | 0.88 | 2.83E-01 | 4.20E-01 |
| *BIRC2* | 106.17 | 117.87 | 0.90 | 1.24E-01 | 2.15E-01 |
| *TAB3* | 108.13 | 118.56 | 0.91 | 2.39E-01 | 3.61E-01 |
| *TLR4* | 44.53 | 48.64 | 0.92 | 5.24E-01 | 6.86E-01 |
| *TNFSF13B* | 5.21 | 5.68 | 0.92 | 7.13E-01 | 8.41E-01 |
| *NFKBIA* | 63.25 | 66.20 | 0.96 | 7.00E-01 | 8.36E-01 |
| *TNFRSF1A* | 111.85 | 116.86 | 0.96 | 5.80E-01 | 7.41E-01 |
| *CARD14* | 10.67 | 11.07 | 0.96 | 8.61E-01 | 9.56E-01 |
| *IRAK4* | 41.11 | 42.62 | 0.96 | 7.51E-01 | 8.75E-01 |
| *BCL2A1* | 3.87 | 3.99 | 0.97 | 9.29E-01 | 9.56E-01 |
| *BCL10* | 52.06 | 52.48 | 0.99 | 9.28E-01 | 9.56E-01 |
| *TRIM25* | 148.25 | 149.36 | 0.99 | 9.13E-01 | 9.56E-01 |
| *MYD88* | 69.50 | 69.89 | 0.99 | 9.35E-01 | 9.56E-01 |
| *PLCG1* | 116.64 | 116.87 | 1.00 | 9.81E-01 | 9.81E-01 |
| *CSNK2B* | 73.69 | 73.53 | 1.00 | 9.74E-01 | 9.81E-01 |
| *TIRAP* | 24.53 | 24.35 | 1.01 | 9.26E-01 | 9.56E-01 |
| *TAB1* | 31.07 | 30.81 | 1.01 | 9.22E-01 | 9.56E-01 |
| *TRAF3* | 39.44 | 38.99 | 1.01 | 8.97E-01 | 9.56E-01 |
| *TICAM1* | 20.02 | 19.78 | 1.01 | 9.35E-01 | 9.56E-01 |
| *TRAF6* | 40.34 | 38.97 | 1.04 | 6.87E-01 | 8.32E-01 |
| *XIAP* | 167.14 | 160.50 | 1.04 | 4.80E-01 | 6.40E-01 |
| *MAP3K7* | 70.84 | 67.81 | 1.04 | 5.91E-01 | 7.44E-01 |
| *CARD10* | 77.83 | 74.24 | 1.05 | 6.66E-01 | 8.17E-01 |
| *PRKCQ* | 8.71 | 8.28 | 1.05 | 8.07E-01 | 9.16E-01 |
| *LCK* | 15.46 | 14.53 | 1.06 | 8.03E-01 | 9.16E-01 |
| *CSNK2A2* | 30.93 | 28.95 | 1.07 | 6.20E-01 | 7.71E-01 |
| *MALT1* | 101.66 | 94.63 | 1.07 | 3.66E-01 | 5.27E-01 |
| *NFKB2* | 57.81 | 53.57 | 1.08 | 4.53E-01 | 6.13E-01 |
| *DDX58* | 64.89 | 58.92 | 1.10 | 4.14E-01 | 5.77E-01 |
| *RELA* | 77.61 | 69.46 | 1.12 | 1.64E-01 | 2.64E-01 |
| *TRAF5* | 138.13 | 123.43 | 1.12 | 1.54E-01 | 2.58E-01 |
| *NFKB1* | 101.45 | 90.62 | 1.12 | 1.14E-01 | 2.01E-01 |
| *CSNK2A1* | 124.68 | 102.32 | 1.22 | 1.19E-02 | 2.74E-02 |
| *TNFRSF11A* | 115.77 | 94.52 | 1.22 | 1.57E-01 | 2.58E-01 |
| *IKBKG* | 4.38 | 3.53 | 1.24 | 5.30E-01 | 6.86E-01 |
| *TRAF2* | 37.45 | 30.19 | 1.24 | 6.14E-02 | 1.16E-01 |
| *CHUK* | 58.66 | 45.28 | 1.30 | 8.65E-03 | 2.21E-02 |
| *LTBR* | 143.53 | 106.59 | 1.35 | 4.77E-03 | 1.41E-02 |
| *PARP1* | 145.09 | 98.80 | 1.47 | 1.01E-03 | 4.61E-03 |
| *UBE2I* | 94.81 | 63.66 | 1.49 | 2.91E-04 | 1.79E-03 |
| *BCL2L1* | 98.91 | 65.91 | 1.50 | 8.61E-05 | 6.17E-04 |
| *LYN* | 75.53 | 47.47 | 1.59 | 3.17E-03 | 1.04E-02 |
| *TICAM2* | 13.78 | 8.40 | 1.64 | 5.00E-03 | 1.44E-02 |
| *IRAK1* | 139.60 | 83.10 | 1.68 | 6.81E-05 | 6.17E-04 |
| *ICAM1* | 73.09 | 38.28 | 1.91 | 1.05E-03 | 4.61E-03 |
| *IL1B* | 40.36 | 19.60 | 2.06 | 4.36E-03 | 1.34E-02 |
| *PLAU* | 55.45 | 26.68 | 2.08 | 1.78E-03 | 6.87E-03 |
| *CCL4* | 5.65 | 2.07 | 2.72 | 9.69E-03 | 2.35E-02 |
| *PTGS2* | 66.54 | 20.60 | 3.23 | 5.78E-04 | 2.95E-03 |
| *CXCL2* | 51.60 | 10.01 | 5.15 | 1.57E-06 | 7.23E-05 |
| *IL8* | 93.15 | 15.82 | 5.89 | 5.83E-05 | 6.17E-04 |
| *CSNK2A1P* | 1.16 | 0.11 | 10.55 | 2.19E-03 | 7.76E-03 |
